# Factors associated with re-entry to out-of-home care among children in England

**DOI:** 10.1016/j.chiabu.2016.11.012

**Published:** 2016-11-28

**Authors:** Louise Mc Grath-Lone, Lorraine Dearden, Katie Harron, Bilal Nasim, Ruth Gilbert

**Affiliations:** a Administrative Data Research Centre for England, University College London, London, UK; b UCL Great Ormond Street Institute of Child Health, University College London, London, UK; c UCL Institute of Education, University College London, London, UK; d Department of Health Service Research and Policy, London School of Hygiene and Tropical Medicine, London, UK

**Keywords:** Looked after children, Out-of-home care, Re-entry, Permanence

## Abstract

Exiting and re-entering out-of-home care (OHC) is considered a disruption to permanence which may have long-lasting, negative consequences for children due to a lack of stability and continuity. Each year approximately one-third of children in OHC in England exit, but information is lacking on rates of re-entries and associated factors. Using national administrative data, we calculated rates of re-entry among children exiting OHC from 2007 to 2012, identified key child and care factors associated with re-entry using Cox proportional hazards modelling, and developed a simple probability calculator to estimate which groups of children are most likely to re-enter OHC within three months. Between 2007 and 2012 reentries to OHC in England decreased (from 23.3% to 14.4% within one year of exit, *p* < 0.001), possibly due to concurrent changes in the way children exited OHC. Overall, more than one-third of children exiting OHC in 2008 re-entered within five years (35.3%, *N* = 4076), but rates of re-entry varied by child and care characteristics including age, ethnicity, mode of exit, and placement stability. Based on these associated factors, we developed a calculator that can estimate the likelihood of rapid re-entry to OHC for a group of children and could be used by social care practitioners or service planners. Our findings provide insight into which groups of children are most likely to re-enter OHC, who may benefit from additional support or ongoing monitoring.

## 1 Introduction

A central goal of England’s social care system is to ensure that children have permanence (Department for Education, 2015a). This permanence (i.e. emotional, physical, and legal security, stability, and continuity (Department for Education, 2013)) helps children develop and maintain a sense of identity and belonging during childhood and beyond (Thomas, 2013). Most children in the care of the State (who are known as looked-after children) are placed in out-of-home care (OHC), such as with a foster carer or in a children’s group home. OHC can provide permanence to children – through stable, long-term foster care, for example. However, current policy favours achieving permanence in a permanent family setting outside of the OHC system, with a particular focus on adoption (Department for Education, 2016a; Department of Health, 2000).

Each year approximately one-third of children exit OHC (Department for Education, 2015b). When exiting OHC a child can either return home to their birth parents (with or without further supervision from social services), be adopted, or be placed with a guardian via a court order (Boddy, 2013). These legal orders include special guardianship and residence orders which confer differing levels of parental responsibility to a guardian but, unlike adoption orders, do not terminate the birth parents’ rights (Department for Education, 2016b). A subsequent re-entry to OHC is considered a breakdown of permanence for a looked-after child, but rates of re-entry are not well-described. Case series studies among children who returned home to their birth parents have reported that almost half re-enter within two years (Farmer & Wijedasa, 2013) and two-thirds within five years (Farmer & Lutman, 2012). However, government figures (which are based on national administrative data) put the five-year re-entry rate after a return home at 30% (Department for Education, 2013). Since their introduction in 2006, two studies have explored special guardianship and residence order breakdowns using national data, and the five-year re-entry rates are estimated to be 6% and 15%, respectively (Selwyn, Wijedasa, & Meakings, 2014; Wade, Sinclair, Stuttard, & Simmonds, 2014). High rates of adoption breakdown (up to 60% in some age groups) have been reported in the media (Henderson, 2012), but a recently-conducted, large-scale academic study found that just 3.2% of adopted children had re-entered OHC within twelve years (Selwyn et al., 2014).

Re-entry to care is associated with a range of child and care characteristics; for example, one study in England found that children were more likely to re-enter OHC if a previous return home had broken down, or there was inadequate preparation and support after their exit (Farmer & Wijedasa, 2013). Similarly, a study of special guardianship orders found a significant association between breakdown and whether the guardian was the child’s former foster carer or relative (Wade et al., 2014). Most recently, an association between more placement moves while in OHC and an increased likelihood of an adoption, special guardianship or residence order breaking down have been described (Selwyn et al., 2014). Studies in other countries have described associations with re-entry to OHC and the child’s age at exit (Orsi, 2015; White, 2016; Yampolskaya, Armstrong, & Vargo, 2007), ethnicity (Orsi, 2015; Shaw, 2006), having behavioral or health problems (Barth, Weigensberg, Fisher, Fetrow, & Green, 2008; Liao & White, 2014; Testa, Snyder, Wu, Rolock, & Liao, 2015; White, 2016; Yampolskaya et al., 2007), a longer time spent in care (McDonald, Bryson, & Poertner, 2006; Wells & Guo, 1999), placement setting (Carnochan, Rizik-Baer, & Austin, 2013; Lee, Jonson-Reid, & Drake, 2012), and placement stability (Carnochan et al., 2013).

A lack of permanence is associated with negative outcomes for children. For example, a qualitative study of fostered and adopted children found that feelings of insecurity hindered the development of close and trusting relationships with their caregivers (Selwyn & Quinton, 2004). It is however difficult to disentangle the causes and consequences of a lack of permanence: a child’s experience of abuse or neglect before entering OHC is likely to affect their feeling of security as well as relationships with caregivers, for example. Nonetheless, (the sometimes repeated cycles of) exits and re-entries to OHC represent a disruption to permanence for children. It has been suggested that improved provision of social care support to children exiting OHC and their families could potentially reduce the rate of re-entry (Holmes, 2014). In order to identify groups with a high likelihood of re-entry and allocate increasingly scarce resources more efficiently, a thorough understanding of the factors associated with re-entry to OHC is needed. However, this is currently lacking as the majority of the published literature on the topic is from the United States and not applicable to the English context, given the significant differences in population demographics, societal structures, and social care systems. In this study, we aimed to use national administrative data to identify child and care factors associated with re-entry to OHC among children in England. We also sought to develop a simple, online calculator that could be used by social care practitioners to identify groups of children who are most likely to re-enter OHC, and thus may have the greatest need for additional support when exiting care.

## 2 Method

### 2.1 Study extract

Since 1992, data related to children in care in England has been routinely collected from local authorities (local government bodies responsible for delivering children’s social care services) by the Department for Education (DfE) using the Children Looked After return (CLA). This longitudinal, individual-level dataset contains information on child characteristics and episodes of care, including: date of birth, ethnicity, reason a child was looked after, placement type, and reason each episode of care ceased. Children’s care histories are linked over time via a unique identifier; however, complete care histories are only available for one-third of children (namely, those whose day of birth is divisible by three as data was not collected for other children between 1998 and 2003). For further details of the CLA dataset see (Mc Grath-Lone, Harron, Dearden, Nasim, & Gilbert, 2016).

For this study, we derived a CLA extract of children who were placed in OHC for non-respite reasons. We did not include children in voluntary, short-term respite placements as their re-entry to OHC is often planned, at regular intervals (e.g., every weekend) and for respite care for serious chronic health conditions. As such, the initial study extract contained all episodes of care from January 1, 1992 to December 31, 2013 for one-third of children born on or after January 1, 1992 who were placed in OHC for non-respite reasons (*N =* 95,369). Ethical approval was not required for this study as it was a secondary analysis of de-identified administrative data; however, all applications for CLA data are reviewed by an advisory panel at DfE before access to the data is granted.

### 2.2 Describing rates of re-entry to out-of-home care

A limitation of CLA data is that it cannot be used to explore re-entry to OHC among children who are adopted. If an adopted child re-enters the social care system they are assigned a new unique identifier in CLA, which prevents linkage of pre- and post-adoption care histories. It is also not appropriate to use CLA data to explore re-entries to OHC among children who exit care because they are sentenced to custody, as their time to re-entry will be affected by the time they spend in custody. Similarly, it is difficult to interpret exits and re-entries to OHC for older adolescents as independent living (where a young person lives in a bedsit, apartment or other lodgings, either alone or with friends) can be used as either a care placement or mode of exit from care and its use varies across local authorities. We therefore excluded children who exited OHC aged 16 or older or via adoption orders or custodial sentences from our re-entry analysis. For all other children, exits since January 1, 2007 (the year special guardianship and residence orders were introduced) were identified and categorised using CLA codes as per Supplementary Table S1. If a child exited OHC more than once in a calendar year, their first exit in that year was selected as the index exit (*N* = 21,716).

Re-entries to OHC by 31 December 2013 were explored using survival analysis methods. The cumulative proportion of children re-entering OHC by year and type of exit was described using Kaplan-Meier curves. The length of follow-up varied by year of exit, from 6 years for children exiting care in 2007 to 1 year for children exiting care in 2012, and follow-up was censored on a child’s 18th birthday (as they were no longer at risk of the outcome of interest).

### 2.3 Identifying factors associated with re-entry to out-of-home care

Factors associated with re-entry to care were explored using a sub-sample of children who exited OHC in 2008 (*N =* 4076, see Supplementary Fig. S1). This year was selected as it allowed re-entry within a comparatively long follow-up period to be explored, but was after the introduction of special guardianship and residence orders. Cox proportional hazards modelling was used as it is a survival analysis method that allows the hazard or likelihood of an outcome to be estimated while accounting for multiple explanatory variables (e.g., demographic or care characteristics).

A Cox proportional hazards model has two main assumptions: firstly, that censoring is non-informative and secondly, that the hazard of an explanatory variable is proportional (i.e. constant over time). In this study, censoring of follow-up was non-informative as only children who had reached the age of 18 and were no longer at risk of the outcome of interest did not have the full five year follow-up. However, several variables violated the proportionality assumption (i.e. their hazards were not constant and changed over time). For these time-varying variables we used Aalen’s linear hazards model (Buchholz, Sauerbrei, & Royston, 2014) to plot the cumulative regression coefficients against follow-up time and identified three periods over which hazards were proportional: 0–3 months, 3–12 months and 1–5 years (see Supplementary Fig. S2). New dummy variables that had proportional hazards in these time periods were derived and so both key assumptions of Cox proportional hazards model were met in this study.

The association between each explanatory variable and re-entry to OHC was initially assessed using a univariate Cox proportional hazard model. A multivariable model was then created in a stepwise fashion by including all variables associated with re-entry at univariate level (where *p* < 0.10) and removing non-significant variables in turn until only significant factors remained. No significant interactions between explanatory variables were identified and the effect of clustering within local authorities was accounted for by using a shared-frailty Cox proportional hazard model (see Supplementary Table S2 for details of the final model). It was not possible to explore variation in the factors associated with re-entry to OHC at a local authority level due to a lack of power.

### 2.4 Developing a tool to estimate the likelihood of re-entry to out-of-home care

Although estimating the likelihood of re-entry to OHC over a period of years would be useful for long term service planning (e.g. in terms of informing future capacity needs), we felt that a shorter period of time was likely to be more relevant to social care practitioners. In this analysis, we chose to focus on re-entries to OHC within three months to supplement social workers understanding of which groups of children are most likely to rapidly re-enter OHC, as these groups may potentially need closer monitoring or additional support. This short period of time also accounted for more than one-third of all re-entries that occurred within five years (i.e. 37.6%). (Future planned work will focus on developing models for longer periods of time).

To explore which groups of children were most likely to re-enter OHC rapidly, a simplified Cox proportional hazards model that included factors associated with re-entry within three months and that a social worker could be reasonably expected to know about a child was developed. For example, while the average length of a child’s placements was significantly associated with re-entry to OHC this information may not be readily available to a social worker and so time in care was included instead. Bootstrapping (x 1000 repetitions) was used to internally validate the effect sizes of the included variables and the baseline hazard of re-entry to care at three months was estimated. This information was then used to develop a model that estimated the absolute likelihood of rapid re-entry to OHC, rather than a relative hazard.

The discrimination of the model (i.e. its ability to distinguish between children who do and do not re-enter care) was assessed by calculating the Harrell’s c-score and its predictive power was evaluated by measuring the Brier score and area under the curve (AUC) of the receiver operating characteristic curve. Finally, an external dataset of children who exited OHC in 2012 (*N =* 4650) was used to validate the model by evaluating the Brier score, AUC and the agreement between the observed and estimated probability of re-entry (Altman & Royston, 2000). The validated model was then used to create a simple, online calculator that estimates the probability of re-entry to OHC within three months.

## 3 Results

### 3.1 Rates of re-entry to out-of-home care among children in England

**Fig. 1:**
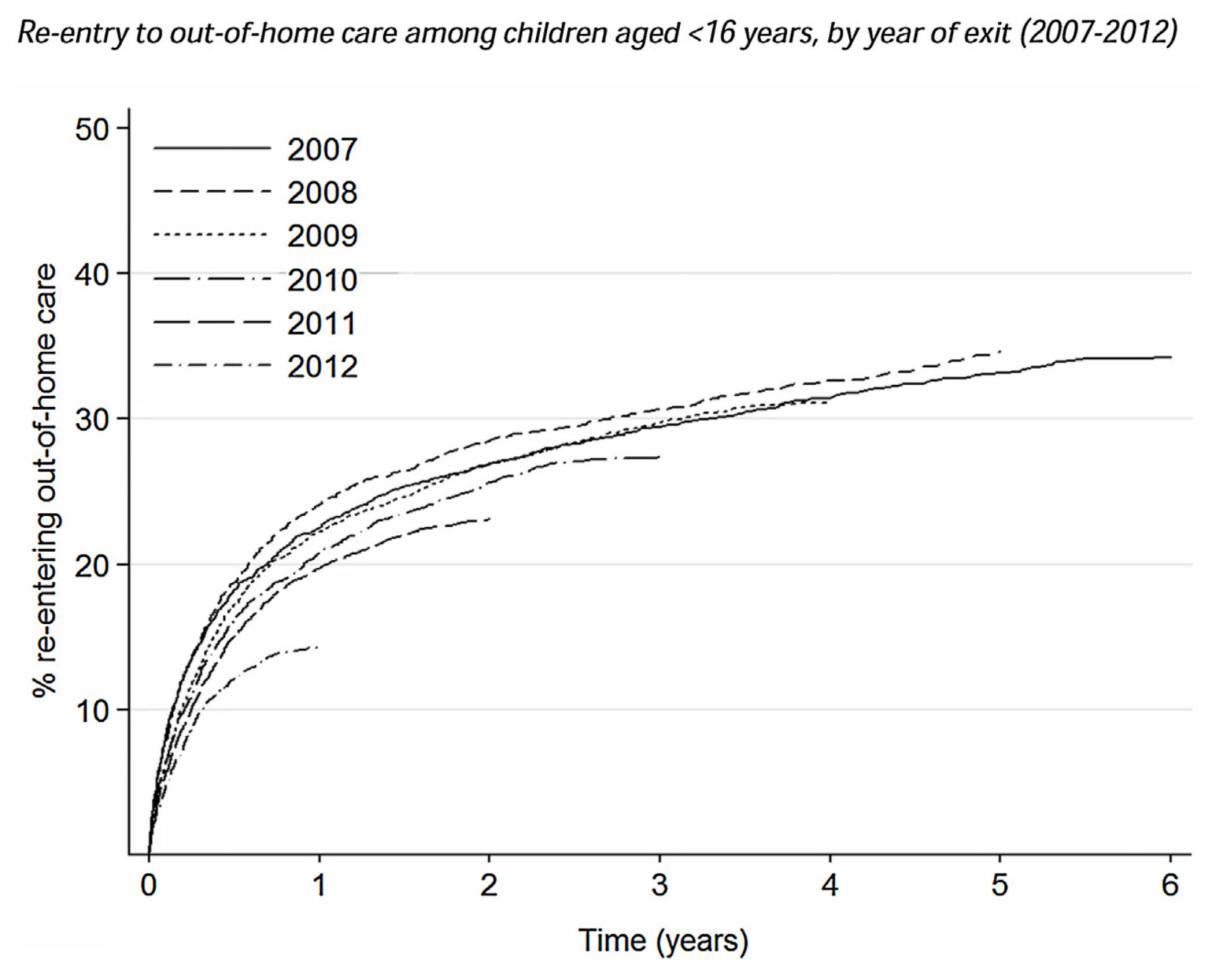
Re-entry to out-of-home care among children aged <16 years, by year of exit (2007–2012). Fig. 1 shows the percentage of children aged <16 when exiting out-of-home care who re-entered by 31st December 2013, stratified by the year they exited. The number of exits (*N*) was 3862 in 2007; 4076 in 2008; 4184 in 2009; 4467 in 2010; 4477 in 2011 and 4650 in 2012. Children who exited out-of-home care because they were adopted or sentenced to custody are not included.

The proportion of children re-entering OHC decreased over time (see Fig. 1). For example, the proportion that re-entered within one year of exit decreased from 23.3% to 14.4% between 2007 and 2012 (*p* < 0.001). However, during this period there were concurrent changes in the type of exit from OHC (see Supplementary Table S3). In particular, there were significant increases in the use of special guardianship and residence orders across all age groups; for example, among children aged 5 to 10 years the proportion exiting care via a special guardianship order increased from 8.2% of exits in 2007 to 19.8% in 2012 (*p* < 0.001).

Detailed characteristics of our sub-sample of children who exited OHC in 2008 (*N =* 4076) are described in Table 1. Overall, 35.3% re-entered OHC within five years of exit (*n =* 1438). On average re-entry occurred within one year of exit (mean: 324 days); however, a fifth of re-entries (*n =* 283) occurred within one month of exit and almost 40% (*n =* 541) within three months.

### 3.2 Factors associated with re-entry to out-of-home care

Among the sub-sample of children who exited OHC in 2008 (*N =* 4076), rates of re-entry to OHC varied significantly by child characteristics such as age at exit and ethnic category (see Table 2). For example, just 26.1% of children of Asian, Black or Other ethnicity re-entered OHC within five years compared to 37.6% of children of White or Mixed ethnicity (*p* < 0.001). Rates of re-entry to OHC within five years also varied by care characteristics: a previous history of being in OHC, placement in group care rather than foster care and being in care voluntarily (i.e. care was not court mandated) were associated with higher rates. In contrast, being placed with a relative (i.e. kin care), longer placements and fewer placement changes were associated with lower rates of re-entry. Re-entry to OHC within five years also varied by the type of exit from care, from 40.5% of children who were returned home to 4.2% of those exiting via a special guardianship order.

Adjusting for other factors, children aged 11 to 15 years when exiting care were more likely than younger children to re-enter within five years (Table 3, HR_adj_: 1.49; 95%CI: 1.27-1.76, *p* < 0.001). Similarly, children of White or Mixed ethnicity were more likely to re-enter OHC compared to children of Asian, Black or Other ethnicity (HR_adj_: 1.50; 95%CI: 1.27-1.76, *p* < 0.001). A consistent association with a previous history of OHC and number of placement changes was also evident. Children who had already exited and re-entered OHC were 44% more likely to re-enter within five years as children exiting care for the first time. Those who had experienced five or more placement changes while in OHC were 56% more likely to re-enter compared to children who had not changed placement.

Other care characteristics were also associated with re-entry to care but had time-varying effects. For example, being in voluntary care rather than court-mandated care was associated with a higher probability of re-entry to OHC; however, the level of increased likelihood diminished over time from 83% in the three months following exit to 47% between one and five years after exit. Similarly, longer placements were associated with lower likelihood of re-entry but the strength of this association decreased over time. The effect of the reason a child was in care also varied over time: children who were in care due to disability were more likely to re-enter care in the long term (i.e. 1–5 years following exit) but there were no significant associations with earlier re-entries (i.e. within three months or 3–12 months). Children in care due to family stress, dysfunction or low income were more likely to re-enter care in the short term (i.e. within three months) and those in care due to absent parenting were consistently less likely to re-enter care within the five year follow-up period. Accounting for other factors, children who were placed with their parents had a higher likelihood of re-entering OHC than those who were returned home throughout the five year follow-up period. Conversely, children who exited via special guardianship or residence orders were consistently less likely to re-enter care.

### 3.3 Estimating the likelihood of re-entering out-of-home care

The model for our probability calculator used baseline risk and proportional hazard ratios to estimate an average likelihood of re-entry to OHC within three months based on the following group characteristics: age group at exit, ethnic category, reason the child was in OHC, had the child exited OHC previously, length of current episode of OHC, whether the child was in voluntary or court-mandated care and the mode of exit from care (e.g. return home, residence order, etc.). This estimation model to calculate the likelihood of rapid re-entry to OHC had a Harrell’s c-score of 0.79 and AUC of 0.78 which indicated good discrimination between children who did and did not re-enter OHC (both measures can range from 0.5 to 1.0, where 1.0 is perfect discrimination). Calibration of the model was also good with a Brier score of 0.11 (which ranges from 0 to 1, where 0 is a perfect prediction).

**Fig. 2:**
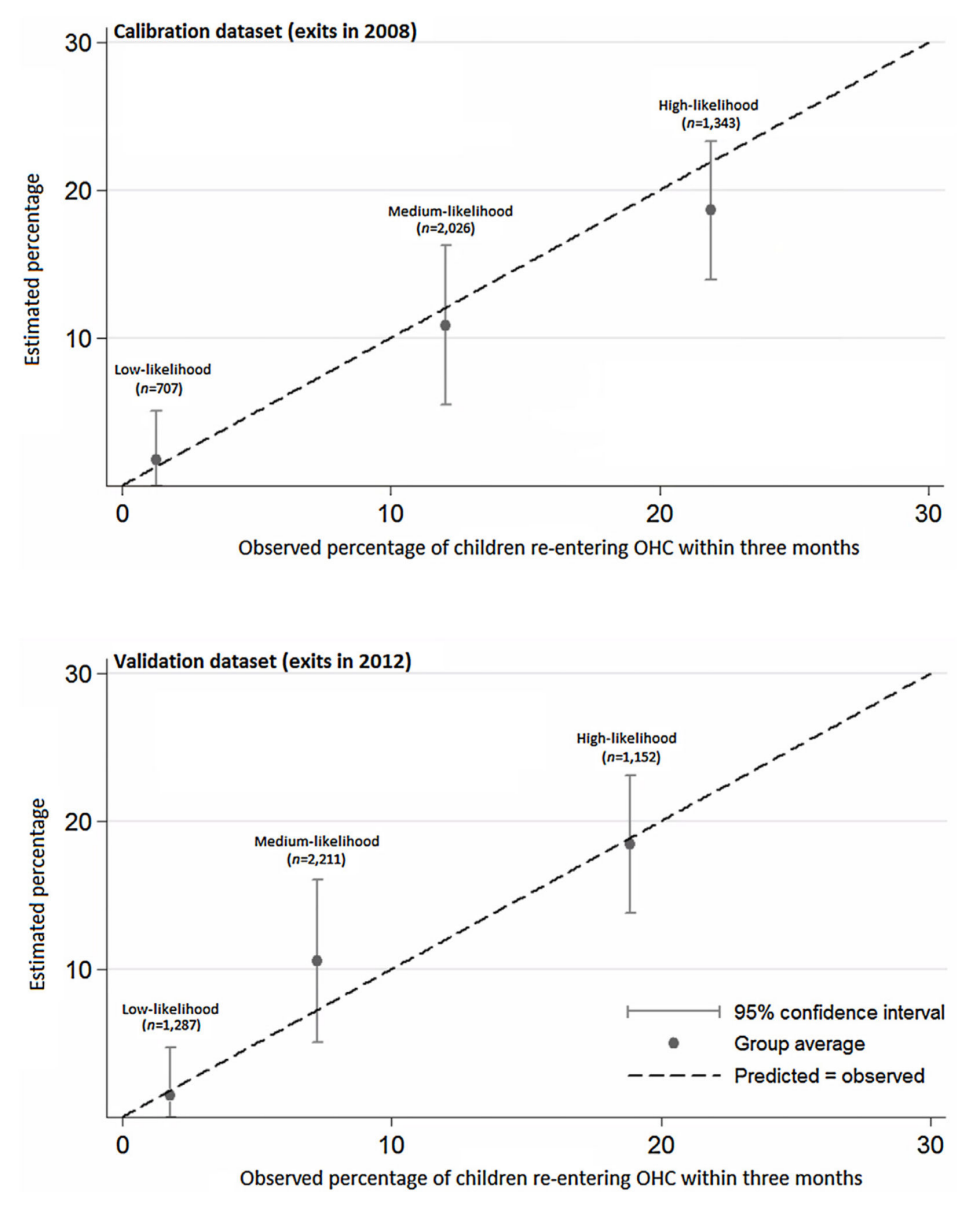
Observed versus estimated percentage of children exiting out-of-home care in 2008 and 2012 who rapidly re-enter. Fig. 2 shows the actual observed percentage of children who re-entered out-of-home care (OHC) within three months versus the percentage estimated by our model for children who exited in 2008 (calibration dataset*, N* = 4076) and 2012 (validation dataset, *N* = 4650). Children were grouped as *low-*, *medium-* or *high-likelihood* based on their demographic and care characteristics (detailed in Supplementary Table S4).

This estimation model calculated a 10% likelihood of re-entry for the “average” group of children (i.e. one with the most common demographic and care characteristics from Table 1). However, the likelihood of re-entry estimated by our model varied between groups from <1% to 29.4% depending on its characteristics (interquartile range: 7.6% to 16.8%). Based on the distribution of likelihood, three categories were created: *low-* (<5%, which included approximately the lowest quartile of children in terms of likelihood), *medium-* (5–15%) and *high-likelihood* (>15%, which included the highest quartile). Fig. 2 illustrates that in the calibration dataset (i.e. children who exited OHC in 2008) there was very good agreement between the likelihood of re-entry to OHC estimated by our model and the actual proportion of children in each category that re-entered OHC within three months.

When the estimation model was applied to a validation dataset of children who exited care in 2012, the Brier score was 0.07, the AUC was 0.75 and again there was good agreement between the estimated likelihood of re-entry to OHC and the actual proportion of each group that re-entered OHC, particularly for the *low-* and *high-likelihood* groups (see Fig. 2). Based on this validated estimation model, we then created a simple, online tool that could be used to calculate a group’s likelihood of re-entering OHC within three months, based on selected demographic and care characteristics. A beta version of the probability of re-entry calculator developed as part of our study is available at https://louisemcgrathlone.com/tools/.

Overall, 17.4% of children who exited care in 2008 (*n =* 707) were categorised as *low-likelihood* for re-entering care within three months, 49.7% (*n =* 2026) as *medium-likelihood* and 32.9% (*n =* 1343) as *high-likelihood*. The estimated rates of re-entry for each group from our probability calculator were <1%, 10.9% and 18.6% respectively, and the actual observed rates were <1%, 12.0% and 21.9%. Older children, those of White or Mixed ethnicity and those in care due to a disability were over-represented in the *high-likelihood* group (see Supplementary Table S4). For example, 71.0% of children in the *high-likelihood* group were aged 11–15 compared to 39.8% of the overall population (*p* < 0.001). Similarly, children who had been in care for longer, in court-mandated care, were exiting care for the first time and who exited via a special guardianship or residence order were over-represented in the *low-likelihood* group. For example, 45.0% of children in the *low-likelihood* group left care through a special guardianship order compared to just 8.3% of the overall population (*p* < 0.001).

Among children who exited care in 2012, 27.7% of children (*n =* 1287) were categorised as *low-likelihood* for re-entering OHC within three months, 47.5% (*n =* 2211) as *medium-likelihood* and 24.8% (*n =* 1152) as *high-likelihood.* The estimated rates of re-entry for each group were 1.4%, 10.5% and 18.4% respectively, and the actual observed rates were 1.7%, 7.2% and 18.8%.

## 4 Discussion

Between 2007 and 2012 the rate of re-entry to OHC among children in England decreased. Results from the probability calculator indicated a change over time in the profile of children exiting OHC: the proportion of children identified as *high-likelihood* for rapid re-entry decreased from one in three children exiting care in 2008 to one in four in 2012 (32.9% vs. 24.8%, *p* < 0.001). Overall, more than one-third of children exiting OHC in 2008 re-entered within five years. However, rates of re-entry varied by child and care characteristics with higher rates associated with older age when exiting OHC, being of White or Mixed ethnicity, returning to parents on exit, and shorter average placement length.

One limitation is that our analyses do not include the small proportion of children (6.3%) who left care aged 16 or 17. As a result, the overall rate of re-entry we calculated is likely to be an underestimation for the total child population in England. Our analyses also could not include children who were adopted as it is not possible to link pre- and post-adoption records of care. Furthermore, limitations in the range and detail of information collected in the CLA dataset meant that we could not distinguish between planned and unplanned exits and re-entries; nor could our analyses account for variation in important parental or child risk factors for re-entry (such as type of abuse, family composition, mental or physical health conditions, exposure to violence, substance misuse, etc.). A strength of our analysis is that we used data for the whole of England with long-term follow up from 1992 to 2013 and included children who returned home, were placed with their parents or left care via a legal order (in comparison to other studies based on sub-national samples or focused on one mode of exit only (Farmer & Lutman, 2012; Farmer & Wijedasa, 2013; Wade, Biehal, Farrelly, & Sinclair, 2010; Wade et al., 2014)). Furthermore, our survival analysis incorporated time-varying hazards and provided more detailed descriptions of the influence of child and care factors on re-entry than other studies that assume proportionality throughout the follow-up period. The key strength of our study is the practical application of our findings: we developed a simple, online calculator that can be used by service planners and social care practitioners to estimate which groups are most likely to rapidly re-enter OHC.

The one in eight children (13.0%) who exited care via a special guardianship or residence order were least likely to re-enter OHC (4.2% and 8.9% within five years, respectively). These estimates of breakdown were slightly lower than those described by (Selwyn et al., 2014), most likely because older adolescents were not included in our sample. Nonetheless, our findings provide further evidence for comparatively lower rates of breakdown associated with special guardianship orders, which may be useful for policy makers and service providers.

As well as mode of exit, placement stability and lifetime experiences of care were important factors associated with rates of re-entry. For example, we found that the total number of placement changes and the average placement length were more significant predictors than the total time spent in care. Children were less likely to re-enter OHC within five years if their placements lasted nine months or longer on average (though the strength of this effect diminished over time). Whereas early instability in care (i.e. two or more placement moves during the first 100 days) had been associated with increased likelihood of re-entry to care in other studies (Akin, 2011), it was not a significant factor in our analyses. This suggests that initial difficulties achieving placement stability may be negated in the long-term with consistent, stable care.

A previous exit and re-entry to OHC was also strongly and consistently associated with an increased hazard of another re-entry. Although the proportion of children who had experienced repeated entries to OHC was relatively small (16.6%), almost half the group re-entered within five years and so they represent a group that could be targeted for additional support. Currently, official government statistics and reports tend to focus on experiences of care during a 12-month period (Department for Education, 2015a), but our findings highlight the importance of taking a longer term view when analysing data related to looked-after children. To ensure the best and most robust evidence base for guiding policy and practice development, analyses should take a longitudinal, life course approach that accounts for experiences of OHC throughout childhood. In particular, such analyses of adoption breakdown could serve as a valuable evidence base given the current focus on increasing the number and speed of adoptions in England (Department for Education, 2012). Adoption breakdown could not be explored in this study due to limitations of the administrative dataset but may be possible in the future as information on re-entry to OHC following adoption has been collected in the CLA dataset since 2013. However, as adoption appears to be a key government policy further work is urgently required to determine how retrospective linkage to enable long-term follow-up could be achieved.

Other care characteristics (such as placement setting or being placed with a relative) did not significantly affect re-entry to OHC, but the context of a child’s entry to the care system did. Although the majority of children (53.7%) enter OHC for reasons of abuse or neglect, more than a quarter of entries (28.0%) were due to family dysfunction, acute stress or low income. These children had the highest rate of re-entry (43.0% within five years), were significantly more likely to re-enter OHC within three months of exit, and more than 80% of re-entries in this group were for the same reason (with a further 12.0% returning to OHC due to abuse or neglect). This suggests that some children may be returning home before the issues that led them to enter OHC have been resolved. Children who were placed in care voluntarily (rather than under a court order) were also more likely to re-enter OHC. One possible explanation for this observed association is that parents can withdraw consent for a voluntary care placement and so it is likely that a proportion of these exits will have received less professional scrutiny and may not have met thresholds for exits that would be required for court-mandated OHC. However, the higher rate of re-entry associated with voluntary placements may also be due to increased use of “trial periods” at home before permanent exits from OHC. As such, it is difficult to interpret the increased likelihood of re-entry to OHC for children on voluntary placements without being able to distinguish between planned and unplanned exits. There is however potential for further work in this area as this information has been collected in the CLA dataset since 2014.

Research that describes factors associated with re-entry to OHC can help social care practitioners to identify groups of children that may require additional support or closer monitoring when exiting care. However, much of the published research on this topic presents results in the form of hazard ratios which can be difficult to interpret meaningfully, particularly if there is no indication of the absolute likelihood of re-entry. In healthcare, risk score calculators are frequently used to incorporate statistical associations from research into clinical practice and service planning, but their use in social care is far more limited. To our knowledge, our online calculator is the first that can be used to estimate the likelihood of rapid re-entry to OHC within three months which account for more than 40% of re-entries. This simple tool could be used by social care practitioners to explore which groups of children are most likely to re-enter OHC and may need support to reduce the likelihood of re-entry. There are also implications for service providers who could gain greater understanding of the profile of their child population. To aid service planning the number of children who are likely to return to OHC within three months could be estimated by calculating the proportion of the population in each likelihood category and their average probability of re-entry. Work to expand our probability calculator to estimate the likelihood of re-entry to OHC over longer periods of time (up to five years) is currently ongoing and may be useful for longer term service and strategy planning. However, it is important to note that unlike risk score calculators in a healthcare context, that may be used to guide treatment decisions, the tool we have developed is not designed or intended to be used for individual care planning or decision-making. The purpose of the tool is to supplement social care practitioners understanding of which groups of children are most likely to rapidly re-enter OHC. The likelihood of rapid re-entry to OHC that is estimated by our model is based on a limited number of group-level characteristics from a national population and results cannot and should not be extrapolated below this level.

Permanence for children exiting OHC in England appears to be improving, as evidenced by falling rates of re-entry between 2007 and 2013. The drivers of this decrease over time require further exploration, but changes in the risk profile of children placed in OHC may be a contributing factor. For example, increasing rates of entry to and lengths of stay in OHC (Mc Grath-Lone, Dearden, Nasim, Harron & Gilbert, 2016) could indicate that thresholds for entering and exiting OHC have changed over time. Children entering and exiting OHC may represent less challenging cases which could account for the lower rates of re-entry observed. Given the significantly lower rates of re-entry associated with special guardianship and residence orders, their increased use may also have contributed to the overall decrease in rates of re-entry over time. As such, these legal orders appear to represent a positive strategy for achieving permanence for vulnerable children. However, local variation in their structure and uptake (Wade et al., 2014) must be acknowledged, as well as the element of selection associated with their use – not all children in care will be able to achieve (or want) this type of care arrangement and legal permanence. Furthermore, while differences in available demographic and care characteristics between children who return home and who exit via these legal orders were controlled for in this analysis, it is likely that there are other differences in child or parental risk factors not recorded in the CLA dataset that may account for some of the variation in the observed rates or re-entry. To fully understand the effectiveness of OHC and arrangements for exiting the OHC system, rigorous comparative studies (which are currently lacking in the evaluation of OHC interventions (Maclean, Sims, O’Donnell & Gilbert, 2016)) are required.

Though movements in and out of the care system are considered a disruption to permanence for already vulnerable children, it is also important to acknowledge that re-entry to OHC is not an intrinsically negative outcome. For example, a series of planned placements with parents that aim to transition a child out of foster care gradually may be preferable to a sudden return home (for both parents and children) (Department of Families, Housing, Community Services and Indigenous Affairs, 2010). Similarly, remaining outside the care system cannot be considered a positive outcome if a child is unhappy or exposed to harm (Fuller, 2005; NSPCC, 2012) and so a re-entry to OHC that is in the best interests of safeguarding and nurturing a child should be viewed positively. However, in a climate of financial cutbacks and growing pressure on social care systems, the challenge is to ensure that avoidable re-entries to OHC (e.g., due to a lack of support or poor planning) are prevented through better targeting of groups who may be highly-likely to re-enter care and more effective use of increasingly scarce resources.

## Supplementary Material

Supplementary data associated with this article can be found, in the online version, at http://dx.doi.org/10.1016/j.chiabu.2016.11.012.

Supplementary data

## Figures and Tables

**Table 1:** Characteristics of children exiting out-of-home care in 2008 (N = 4076).


Child characteristics					
Sex	n	%	Age at exit (years)	n	%
Male	2144	52.6	<1	436	10.7
Female	1932	47.4	1 to 4	1096	26.9
			5 to 10	923	22.6
*Ethnic category*^a^			11 to 15	1621	39.8
White	2896	71.1			
Mixed	378	9.3	Mean	8 years	
Asian	230	5.6	Median	8 years	
Black	465	11.4			
Other (including Chinese)	88	2.2			
Care characteristics at entry					
*Reason for entering OHC*^b^	*n*	%	*In OHC voluntarily?*	*n*	%
Abuse or neglect	2189	53.7	Yes	2546	62.5
Child’s disability	79	1.9	No	1530	37.5
Parental disability	284	7.0			
Family in acute stress	506	12.4	*Type of placement*		
Family dysfunction	614	15.1	Foster care	3599	88.3
Socially unacceptable behavior	184	4.5	Group care	413	10.1
Low income	15	0.4	Other	64	1.6
Absent parenting	205	5.0			
			*Placed with kin at entry?*		
*Previous history of OHC?*			Yes	295	7.2
Yes	678	16.6	No	3781	92.8
No	3398	83.4			
Care characteristics at exit					
*Placement changes*	*n*	%	*In OHC voluntarily?*	*n*	%
None	2456	60.3	Yes	2502	61.4
1 to 4 changes	1518	37.2	No	1574	38.6
5+ changes	102	2.5			
			*Type of placement*		
*Time in OHC*			Foster care	3564	87.4
Mean	297 days		Group care	423	10.4
Median	93 days		Other	89	2.2
<12 months	2103	51.6	*Placed with kin at exit?*		
12+ months	1973	48.4	Yes	638	15.7
			No	3438	84.3
*Average placement length*					
<3 months	2136	52.4	*Type of exit from OHC*^d^		
3–9 months	989	24.3	Returned home	2560	62.8
9+ months	951	23.3	Placed with parents	598	14.7
			Special guardianship	337	8.3
*Early instability of OHC?*^c^			Residence order	190	4.7
Yes	669	16.4	Other	391	9.6
No	3407	83.6			


OHC = out-of-home care.

a Ethnicity was not recorded for 0.5% (*n =* 19).

b Though there may be multiple reasons why a child enters OHC, only one can be recorded in the Children Looked After (CLA) dataset. When more than one applies to a case the highest ordered reason in the list is chosen. For further details of these “category of need” codes please see (Mc Grath-Lone et al., 2016a).

c Early instability of care was defined as more than two placement changes in the first 100 days of care (as per (Akin, 2011)).

d Children returned home are no longer under the supervision of social services, whereas children placed with parents continue to be supervised. Periods of being looked after that ceased for any other reason are recorded as “other” in the CLA dataset.

**Table 2:** Percentage of children who exited out-of-home care in 2008 and re-entered within five years of exit and univariate association in Cox proportional hazard model.

	%	HR	95% CI	*p*-value		%	HR	95% CI	*p*-value
Child characteristics					Care characteristics at exit				
*Sex*					*Placement changes*				
Male	35.8	(ref)			None	32.8	(ref)		
Female	34.7	0.94	0.85–1.04	0.25	1 to 4	35.6	1.22	1.10–1.36	**<0.001**
*Age at exit (years)*					5+	64.7	2.90	2.09–3.62	**<0.001**
<1	31.0	(ref)			*Time in OHC*				
1 to 4	24.5	0.74	0.60–0.91	**0.004**	<12 months	38.7	(ref)		
5 to 11	29.7	0.89	0.73–1.10	0.28	12+ months	31.6	0.87	0.78–0.96	**<0.001**
11 to 15	46.9	1.71	1.41–2.06	**<0.001**	
					*Average placement length*				
*Ethnic category*^a^					<3 months	42.7	(ref)		
Black, Asian or Other	26.1	(ref)			3–9 months	33.9	0.84	0.74–0.95	**0.01**
White or Mixed	37.6	1.63	1.40–1.89	**<0.001**	9+ months	20.1	0.42	0.36–0.50	**<0.001**
Care characteristics at entry					*Early instability of OHC?*^c^				
*Reason for entering OHC*^b^					No	33.9	(ref)		
Abuse or neglect	31.2	(ref)			Yes	42.5	1.77	1.55–2.02	**<0.001**
Child disability	41.8	1.32	0.94–1.86	0.11					
Parental health	37.3	1.13	0.92–1.39	0.25	*Placement category*				
Family stress/dysfunction	43.0	1.47	1.31–1.66	**<0.001**	Family	33.2	(ref)		
Unacceptable behavior	50.5	1.81	1.46–2.25	**<0.001**	Group	52.0	2.07	1.79–2.39	**<0.001**
Absent parenting	16.6	0.43	0.31–0.61	**<0.001**	Other	38.2	1.30	0.92–1.83	0.13
*Previous history of OHC?*					*In OHC voluntarily at exit?*				
No	32.5	(ref)			No	42.0	(ref)		
Yes	49.3	1.85	1.64–2.09	**<0.001**	Yes	24.7	1.52	1.35–1.71	**<0.001**
*In OHC voluntarily at entry?*					*Placed with kin at exit?*				
No	40.6	(ref)			No	38.6	(ref)		
Yes	26.5	1.54	1.37–1.73	**<0.001**	Yes	17.2	0.35	0.29–0.43	**<0.001**
*Type of placement*					*Type of exit from OHC*^d^				
Family or other	34.3	(ref)			Returned home	40.5	(ref)		
Group	43.8	1.09	1.02–1.18	**0.04**	Placed with parents	39.8	1.09	0.77–1.38	0.89
					Special guardianship order	4.2	0.08	0.04–0.13	**<0.001**
*Placed with kin at entry?*					Residence order	8.9	0.17	0.10–0.27	**<0.001**
No	36.4	(ref)			Other	34.0	0.83	0.69–0.99	**0.04**
Yes	20.4	0.46	0.36–0.60	**<0.001**					

OHC = out-of-home care; HR = hazard ratio; CI = confidence interval. Bold denotes significance at level *p* < 0.05. Overall, 4076 children who exited OHC in 2008 were included in the analysis: the *N* for each characteristic in Table 2 is as per *n* in Table 1.

a The assumption of proportional hazards was only met when ethnicity was binarised as ‘White or Mixed’ versus ‘Asian, Black or Other’. Ethnicity was not recorded for 0.5% (*n =* 19).

b Though there may be multiple reasons why a child enters OHC, only one can be recorded in the Children Looked After (CLA) dataset. The highest ordered reason in the list is chosen when more than one applies to a case. As there was no significant difference between the survival curves of children in care due family dysfunction, acute stress or low income, these reasons for entry to OHC were combined.

c Early instability of care was defined as more than two placement changes in the first 100 days of care (as per (Akin, 2011)).

d Children returned home are no longer under the supervision of social services, whereas children placed with parents continue to be supervised. Periods of being looked after that ceased for any other reason are recorded as “other” in the CLA dataset.

**Table 3:** Factors associated with re-entry to OHC among children who exited care in 2008.

	Re-enter within 3 months			Re-enter within 3–12 months			Re-enter within 1–5 years		
Child characteristics	HR_adj_	95% CI	*p*-value	HR_adj_	95% CI	*p*-value	HR_adj_	95% CI	*p*-value
*Age at exit (years)*									
<1	(ref)			(ref)			(ref)		
1 to 4	0.95	0.77–1.18	0.64	0.95	0.77–1.18	0.64	0.95	0.77–1.18	0.64
5 to 11	1.12	0.91–1.39	0.30	1.12	0.91–1.39	0.30	1.12	0.91–1.39	0.30
11 to 15	1.49	1.27–1.76	**<0.001**	1.49	1.27–1.76	**<0.001**	1.49	1.27–1.76	**<0.001**
*Ethnic category*									
Black, Asian or Other	(ref)			(ref)			(ref)		
White or Mixed	1.50	1.27–1.76	**<0.001**	1.50	1.27–1.76	**<0.001**	1.50	1.27–1.76	**<0.001**
Care characteristics at entry	**HR***_adj_*	**95% CI**	***p***-value	**HR***_adj_*	**95% CI**	***p***-value	**HR***_adj_*	**95% CI**	***p***-value
*Reason in OHC*									
Abuse or neglect	(ref)			(ref)			(ref)		
Child disability	1.30	0.75–2.27	0.35	0.88	0.45–1.72	0.70	1.45	1.03–1.78	**0.04**
Parental health	0.90	0.62–1.32	0.58	1.09	0.76–1.56	0.63	1.23	0.87–1.74	0.24
Family stress or dysfunction	1.48	1.22–1.80	**<0.001**	1.17	0.95–1.45	0.14	0.96	0.76–1.21	0.72
Unacceptable behavior	1.09	0.74–1.60	0.66	1.60	1.12–2.29	**0.01**	1.36	0.87–2.13	0.18
Absent parenting	0.54	0.31–0.94	**0.03**	0.44	0.25–0.80	**0.01**	0.35	0.17–0.71	**0.004**
*Previous history of OHC?*									
No	(ref)			(ref)			(ref)		
Yes	1.44	1.26–1.64	**<0.001**	1.44	1.26–1.64	**<0.001**	1.44	1.26–1.64	**<0.001**
Care characteristics at exit	**HR***_adj_*	**95% CI**	***p***-value	**HR***_adj_*	**95% CI**	***p***-value	**HR***_adj_*	**95% CI**	***p***-value
*Average placement length*									
<3 months	(ref)			(ref)			(ref)		
3–9 months	0.46	0.36–0.59	**<0.001**	1.04	0.84–1.29	0.47	1.18	0.93–1.48	0.17
9+ months	0.34	0.25–0.47	**<0.001**	0.51	0.43–0.77	**<0.001**	0.61	0.46–0.83	**0.001**
*Placement changes*									
No changes	(ref)			(ref)			(ref)		
1 to 4 changes	1.03	0.87–1.28	0.63	1.03	0.87–1.28	0.63	1.03	0.87–1.28	0.63
5+ changes	1.56	1.50–1.64	**<0.001**	1.56	1.50–1.64	**<0.001**	1.56	1.50–1.64	**<0.001**
*In OHC voluntarily?*									
No	(ref)			(ref)			(ref)		
Yes	1.83	1.35–2.46	**<0.001**	2.03	1.50–2.76	**<0.0001**	1.47	1.09–1.91	**0.01**
*Type of exit from OHC*									
Returned home	(ref)			(ref)			(ref)		
Placed with parents	6.64	4.58–9.63	**<0.001**	9.72	6.69–14.1	**<0.001**	6.50	4.54–9.29	**<0.001**
Special guardianship order	0.01	0.01–0.03	**<0.001**	0.15	0.05–0.42	**<0.001**	0.26	0.13–0.51	**<0.001**
Residence order	0.15	0.04–0.63	**0.01**	0.40	0.20–0.83	**<0.001**	0.27	0.13–0.58	**0.001**
Other	1.21	0.93–1.58	0.16	0.79	0.57–1.11	0.17	0.57	0.38–0.78	**0.01**

OHC = out-of-home care; HR_adj_ = adjusted hazard ratio; CI = confidence interval. Bold denotes significance at level *p* < 0.05. Three periods of follow-up during which the hazards of explanatory variables were proportional were identified; 0 to 3 months, 3 to 12 months and 1 to 5 years. The corresponding columns in Table 3 present the hazard ratio of re-entry among the population still at risk of re-entry during this period (i.e. excluding children who had already re-entered care). The sample sizes (*N*) was 4076 between 0 and 3 months; 3535 between 3 and 12 months and 3054 between 1 and 5 years. Theta for shared frailty by local authority in the Cox proportional hazards model was 0.07, *p* = 0.001.
