# Supplementary material for: Factors associated with re-entry to out-of-home care among children in England
Source: Child Abuse Negl. Author manuscript; Available in PMC 2018 Oct 26. (PMC6203309; doi:10.1016/j.chiabu.2016.11.012)
Supplement: Supplementary data [file NIHMS80109-supplement-Supplementary_data.pdf]

Supplementary Material

Supplementary Table 1

Identifying exits and re-entries to out-of-home care using codes in Children Looked After dataset

Code	Department for Education description	Exit from OHC?	Exit type	Possible to identify re-entries to OHC?
P1	Placed at home to live with parents, relatives, or other person with parental responsibility, but continues to be subject to a care order.	Yes	Placed with parents [a]	Yes
E4	Returned home to live with parents, relatives, or other person with parental responsibility (not under a residence order or special guardianship order).	Yes	Returned home	Yes
E43	Special guardianship order made to former foster carers.	Yes	Special guardianship order	Yes
E44	Special guardianship order made to carers other than former foster carers.			
E41	Residence order granted.	Yes	Residence order	Yes
E5	Moved into independent living arrangement and no longer looked after: supportive accommodation providing formalised advice/support arrangements (e.g. most hostels, YMCAs, foyers, and care leavers projects).	Yes	Independent living [b]	Yes
E6	Moved into independent living arrangement and no longer looked after: supportive accommodation providing no formalised advice/support arrangements (e.g. bedsit, own flat, living with friends).			
E8	Period of being looked after ceased for any other reason.	Yes	Other	Yes
E11	Adopted – application for an adoption order unopposed.	Yes	Adoption	No [c]
E12	Adopted – consent dispensed with by court.			
E9	Sentenced to custody.	Yes	Sentenced to custody	Yes [d]
E2	Died.	Yes	Died	n/a
E7	Transferred to care funded by Adult Social Services.	Yes	Transferred to adult social services [e]	n/a
E3	Care taken over by another local authority in the UK.	No - care continues in another local authority [f]	n/a	n/a

OHC=out-of-home care. CLA = Children Looked After. [a]Episodes of care that cease due to a child's placement changing from out-of-home accommodation (such as foster care) to "placed with parents" can also be interpreted as an exit from OHC, as the child is returned home but continues to be supervised by the local authority. [b]Independent living is only used for older adolescents, typically after the age of 16. [c]Children who are adopted are assigned new child identifiers, which prevents linkage of pre- and post-adoption records of care. [d]Time to re-entry will be influenced by length of stay in custody, which is not recorded in the CLA dataset. [e]Generally, only children that require ongoing residential accommodation transfer to adult social services. This transfer usually occurs at age of 18. [f]As the main identifier in CLA dataset (child ID) is local authority specific, it is not possible to link records of care across these boundaries.

Supplementary Table 2

Hazard ratios during development of the final multivariate Cox proportional hazards model

Model number (variables included)		M1 (Exit type)			M2 (M1 + child characteristics)			M3 (M2 + care characteristics at entry)			M4 (M3 + care characteristics at exit)		
Time since exit (months)		0-3	3-12	12-60	0-3	3-12	12-60	0-3	3-12	12-60	0-3	3-12	12-60
Type of exit	Returned home	(ref)	(ref)	(ref)	(ref)	(ref)	(ref)	(ref)	(ref)	(ref)	(ref)	(ref)	(ref)
	Placed with parents	**1.42**	**3.18**	**0.85**	**2.92**	**4.81**	**4.47**	**3.05**	**4.87**	**4.17**	**6.64**	**9.72**	**6.50**
	Special guardianship order	**0.01**	**0.06**	**0.16**	**0.01**	**0.07**	**0.17**	**0.01**	**0.07**	**0.16**	**0.01**	**0.15**	**0.26**
	Residence order	**0.06**	**0.23**	**0.21**	**0.06**	**0.24**	**0.21**	**0.07**	**0.25**	**0.21**	**0.15**	**0.40**	**0.27**
	Other	1.12	**0.72**	**0.55**	1.04	**0.68**	**0.51**	1.07	**0.69**	**0.51**	1.21	0.79	**0.57**
Age group at exit (years)	<1 year				(ref)	(ref)	(ref)	(ref)	(ref)	(ref)	(ref)	(ref)	(ref)
	1 to 4 year				0.86	0.86	0.86	**0.80**	**0.80**	**0.80**	0.95	0.95	0.95
	5 to 11 years				1.03	1.03	1.03	0.60	0.60	0.60	1.12	1.12	1.12
	11 to 15 years				**1.57**	**1.57**	**1.57**	**1.31**	**1.31**	**1.31**	**1.49**	**1.49**	**1.49**
Ethnic category	Black, Asian or Other				(ref)	(ref)	(ref)	(ref)	(ref)	(ref)	(ref)	(ref)	(ref)
	White or Mixed				**1.64**	**1.64**	**1.64**	**1.44**	**1.44**	**1.44**	**1.50**	**1.50**	**1.50**
Reason in OHC	Abuse or neglect							(ref)	(ref)	(ref)	(ref)	(ref)	(ref)
	Child disability							1.21	0.85	1.35	1.30	0.88	**1.45**
	Parental health							0.96	1.17	1.26	0.90	1.09	1.23
	Family stress or dysfunction							**1.55**	**1.24**	0.99	**1.48**	0.17	0.96
	Unacceptable behavior							1.12	**1.63**	1.41	1.09	1.60	1.36
	Absent parenting							**0.54**	**0.47**	**0.34**	**0.54**	**0.44**	**0.35**
Previous history of OHC?	No							(ref)	(ref)	(ref)	(ref)	(ref)	(ref)
	Yes							**2.06**	**2.06**	**2.06**	**1.44**	**1.44**	**1.44**
Placement changes	No changes										(ref)	(ref)	(ref)
	1 to 4 changes										1.03	1.03	1.03
	5+ changes										**1.50**	**1.50**	**1.50**
Average placement length	<3 months										(ref)	(ref)	(ref)
	3-9 months										**0.46**	1.04	1.18
	9+ months										**0.34**	**0.51**	**0.61**
In care voluntarily?	No										(ref)	(ref)	(ref)
	Yes										**1.83**	**2.03**	**1.47**

OHC=out-of-home care. Bold denotes significance at level $p < 0.05$. Three periods of follow-up during which the hazards of explanatory variables were proportional were identified; 0 to 3 months, 3 to 12 months and 1 to 5 years. The corresponding columns in Supplementary Table 2 present the hazard ratio of re-entry among the population still at risk of re-entry during this period (i.e. excluding children who had already re-entered care). The sample sizes for each period are as follows; 0 and 3 months *N*=4,076; 3 to 12 months *N*=3,535; 12 to 60 months *N*=3,054.

Supplementary Table 3

Percentage of exits from out-of-home care (2007-2012), by age group at exit

	Year of exit						Change	
	**2007**	**2008**	**2009**	**2010**	**2011**	**2012**	**over time**	***p*-value**
**<1 year (*N*)**	*469*	*466*	*493*	*503*	*514*	*595*		
Returned home	51.8	54.9	52.9	50.5	50.6	42.2	-9.6%	**0.002**
Placed with parents	20.7	20.2	19.1	22.5	27.0	23.9	+3.2%	0.22
Adopted	9.8	6.4	4.9	4.0	4.1	6.4	-3.4%	**0.04**
Special guardianship order	6.6	5.8	7.7	7.4	7.6	14.1	+7.5%	**< 0.001**
Residence order	4.5	5.2	6.5	6.6	4.7	8.1	+3.6%	**0.02**
Other	6.6	7.5	8.9	9.1	6.0	5.4	-1.2%	0.41
**1 to 4 years (*N*)**	*1,675*	*1,865*	*1,821*	*2,012*	*2,220*	*2,350*		
Returned home	31.5	31.8	35.3	31.4	27.2	25.4	-6.1%	**< 0.001**
Placed with parents	11.5	10.7	9.0	10.3	10.8	9.9	-1.7%	0.10
Adopted	39.9	41.2	38.7	35.4	37.5	39.0	-1.0%	0.56
Special guardianship order	8.1	8.1	8.7	13.3	15.7	17.4	+9.3%	**< 0.001**
Residence order	4.4	3.9	4.3	4.8	5.7	5.9	+1.5%	**0.03**
Other	4.5	4.2	4.0	4.8	3.2	2.4	-2.1%	**< 0.001**
**5 to 10 years (*N*)**	*1,238*	*1,169*	*1,239*	*1,326*	*1,325*	*1,496*		
Returned home	46.0	48.8	46.2	50.3	44.5	39.7	-6.3%	**< 0.001**
Placed with parents	13.2	10.7	11.9	10.0	12.4	9.6	-3.7%	**0.003**
Adopted	21.8	21.0	22.0	18.6	18.3	19.3	-2.5%	0.10
Special guardianship order	8.2	9.3	9.3	11.8	15.2	19.8	+11.6%	**< 0.001**
Residence order	4.8	5.0	4.4	5.0	5.4	8.0	+3.2%	**< 0.001**
Other	6.0	5.0	6.2	4.4	4.2	3.7	-2.3%	**0.01**
**11 to 15 years (*N*)**	*1,485*	*1,643*	*1,647*	*1,625*	*1,526*	*1,470*		
Returned home	66.3	69.3	70.0	69.7	72.2	69.6	+3.3%	0.06
Placed with parents	13.5	11.0	10.0	10.5	8.7	9.7	-3.7%	**0.001**
Adopted	1.3	1.5	0.9	1.2	0.8	1.2	-0.1%	0.81
Special guardianship order	3.6	3.0	4.4	4.4	5.9	6.5	+2.9%	**< 0.001**
Residence order	1.3	2.1	1.9	2.8	2.5	3.4	+2.1%	**< 0.001**
Independent living	0.6	0.9	0.5	0.5	0.5	0.6	N/A	0.99
Other	13.4	12.2	12.4	11.0	9.4	9.0	-4.4%	**< 0.001**

Children placed with parents continue to be supervised by a local authority, children returned home do not. Periods of being looked after that cease for any other reason are recorded as “other” in the Children Looked After return. The total number of exits (*N*) was 4,867 in 2007, 5,143 in 2008, 5,200 in 2009, 5,466 in 2010, 5,585 in 2011 and 5,911 in 2012.

Supplementary Table 4

Percentage of children exiting out-of-home care in 2008 and 2012 with selected characteristics, by likelihood of rapid re-entry

**Year of exit**		**2008**			**2012**	
**Likelihood-group**	**Low (*N*=707)**	**Medium (*N*=2,026)**	**High (*N*=1,343)**	**Low (*N*=1,287)**	**Medium (*N*=2,211)**	**High (*N*=1,152)**
Age at exit (years)						
<1	6.7	13.4	8.8	10.2	15.0	8.1
1 to 4	59.8	32.2	1.5	54.0	32.8	1.2
5 to 10	23.5	24.9	18.8	27.3	27.5	21.6
11 to 15	10.0	29.5	71.0	8.5	24.7	69.1
Ethnic category [a]						
White	65.1	64.4	84.2	72.6	67.0	81.7
Mixed	10.0	10.1	7.7	10.1	10.0	9.3
Asian	6.8	7.5	2.2	5.1	6.7	2.4
Black	15.7	14.5	4.5	9.4	12.7	4.7
Other (including Chinese)	2.4	3.0	0.7	2.3	2.5	0.8
Reason for entering OHC [b]						
Abuse or neglect	67.8	60.1	36.7	70.2	65.7	43.7
Child's disability	0.1	0.2	5.6	0.0	0.4	5.3
Parental disability	8.5	8.1	4.4	6.8	5.6	2.5
Family in acute stress	5.1	9.3	21.1	5.5	7.5	17.8
Family dysfunction	9.5	11.0	24.0	13.0	14.0	26.6
Socially unacceptable behavior	1.0	3.8	7.5	0.6	2.7	3.9
Low income	0.4	0.3	0.5	0.4	0.2	0.2
Absent parenting	7.6	7.3	0.2	3.6	4.0	0.1
Previous history of OHC?						
Yes	87.6	88.1	74.1	90.3	90.0	77.1
No	12.4	11.9	24.9	9.7	10.0	22.9
In OHC voluntarily?						
Yes	11.0	54.9	97.7	15.1	54.2	97.1
No	89.0	45.1	2.3	84.9	45.8	2.9
Average placement length						
<3 months	6.7	32.5	64.7	3.5	27.2	61.9
3-9 months	17.3	13.7	2.2	16.7	14.6	2.5
9+ months	76.0	53.8	33.1	79.8	58.2	35.6
Type of exit from OHC [c]						
Returned home	20.1	62.0	86.5	10.3	59.7	88.0
Placed with parents	7.9	26.0	1.2	4.0	26.3	2.3
Special guardianship	45.0	0.9	-	61.9	3.9	-
Residence order	21.9	1.7	-	21.9	3.3	0.2
Other	5.1	9.4	12.3	1.9	6.8	9.5

Supplementary Table 4 shows selected characteristics (i.e. those included in the predictive model) of children in the calibration and validation datasets. OHC=out-of-home care. [a]Ethnicity was not recorded for 0.5% (*n*=19) of the children exiting OHC in 2008 and 1.0% (*n*=45) of children exiting in 2012. [b]Though there may be multiple reasons why a child enters out-of-home care, only one can be recorded in the Children Looked After (CLA) dataset. When more than one applies to a case the highest ordered reason in the list is chosen. For further details of these "category of need" codes please see (Mc Grath-Lone et al, 2016). [c]Children returned home are no longer under the supervision of social services, whereas children placed with parents continue to be supervised. Periods of being looked after that ceased for any other reason are recorded as "other" in the CLA dataset.

Supplementary Figure 1

Flow chart of study sample creation from Children Looked After data

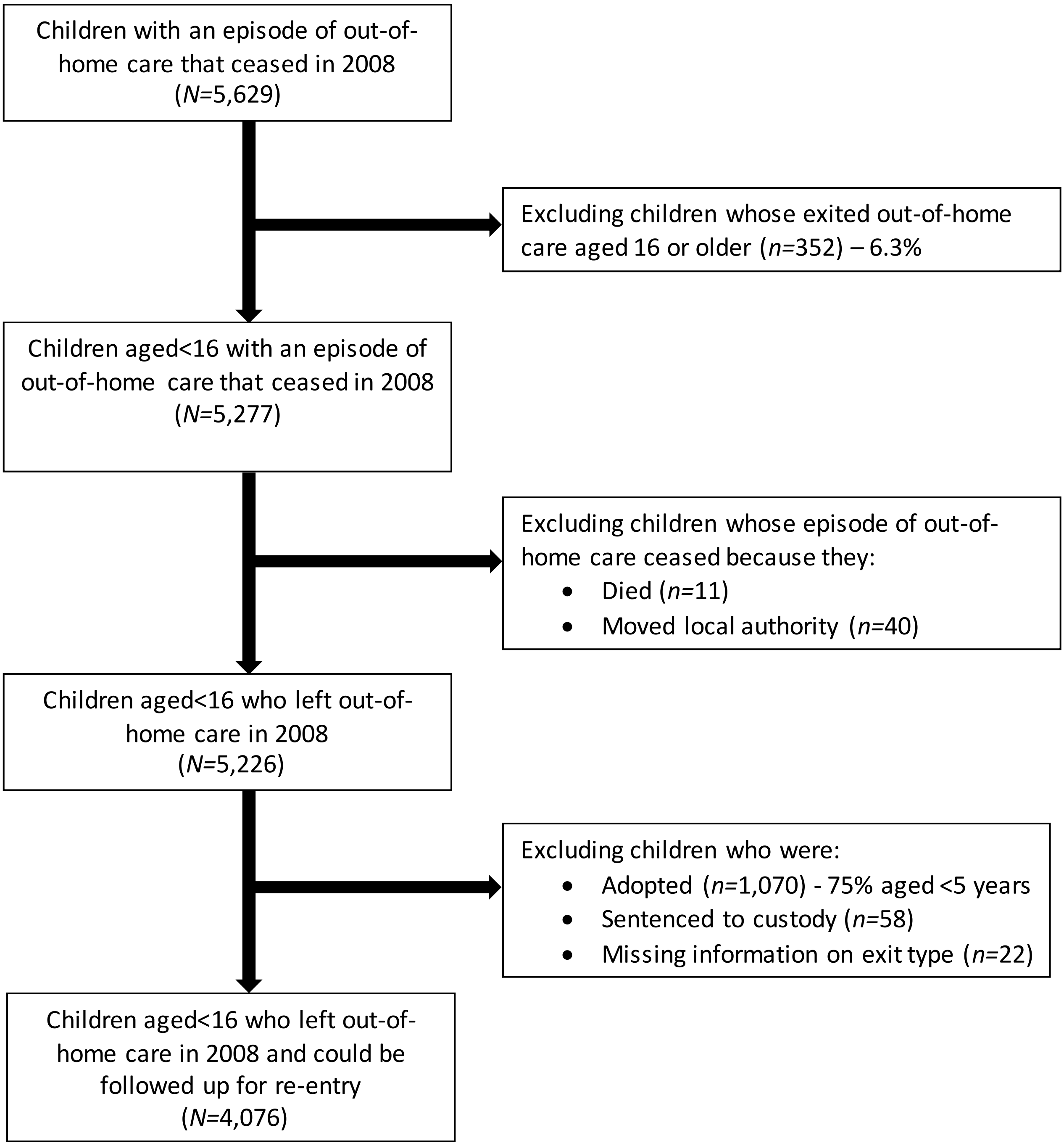

Supplementary Figure 2

Cumulative regression coefficient indicating time-varying effect

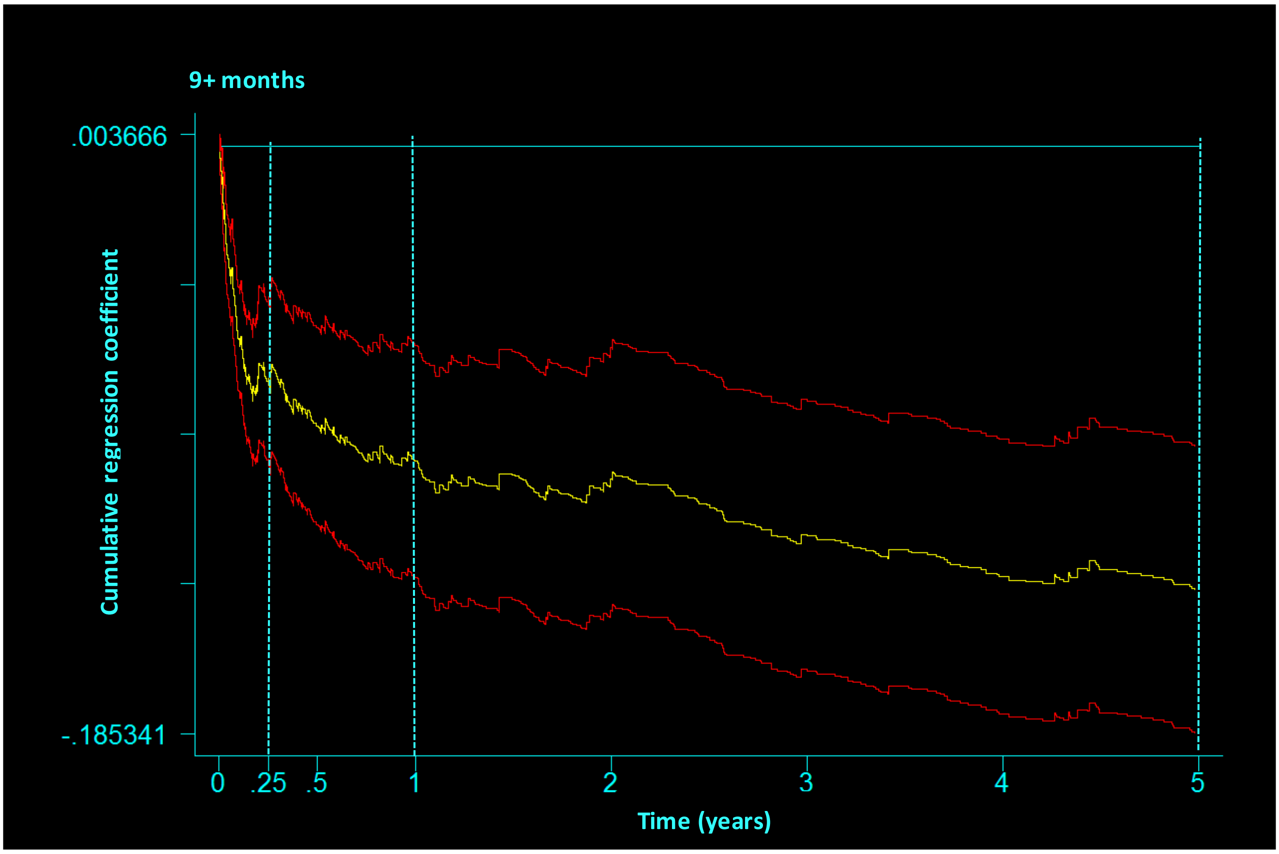

Supplementary Figure 2 shows the cumulative regression coefficient (yellow line) with 95% confidence intervals (red lines) for the explanatory variable "average placement length" of nine months or longer using Aalen's linear hazard model. From the plot, three periods during the five year follow-up for which there are time-varying proportional hazards for the explanatory variable were identified. A strong negative effect was evident from 0 to 3 months, between 3 months and 1 year the strength of the effect diminished and after one year decreased further. As a result, three variables were created to capture the varying relationship over time between a child's average placement length and re-entry to out-of-home care.
